# Adolescent’s self-reported weight and its association with media impact on decision to lose weight and body thinness perception

**DOI:** 10.1038/s41598-022-09909-z

**Published:** 2022-04-08

**Authors:** Hazzaa M. Al-Hazzaa, Balqees A. Al-Awadhi, Yousef A. Al-Dashti, Fahhad A. Alajmi, Fawaz D. Almansour, Ahmed R. Al-Haifi

**Affiliations:** 1 Professor Emeritus, Lifestyle and Health Research Center, Health Sciences Research Center, Princess Nourah Bint Abdulrahman University, Riyadh, 11671 Saudi Arabia; 2 Department of Food and Nutrition Science, College of Health Sciences, PAAET, Showaikh, Kuwait; 3 Department of Food Science and Nutrition, College of Life Sciences, Alshedadeya Campus, University of Kuwait, Kuwait City, Kuwait

**Keywords:** Obesity, Developmental biology, Medical research, Risk factors

## Abstract

This study investigated the accuracy of self-reported weight among Kuwaiti adolescents and the associations of self-reported weight and calculated BMI with the impact of media use on adolescent’s decision to lose weight and body thinness perception. A total of 706 Kuwaiti adolescents (363 females) 15–18 year-olds were randomly selected from schools. Weight and height were self-reported by the adolescents and measured by the researchers. A specifically designed questionnaire reported the impact of media use on adolescent’s decision to lose weight and body thinness perception. There were significant (p < 0.001) relationships between measured and reported weight (r = 0.947), height (r = 0.777), and calculated BMI (r = 0.623). Intraclass correlation coefficients (95% CI) between self-reported and measured weight and height were 0.973 (0.968–0.977) and 0.867 (0.839–0.891), respectively. The mean differences between self-reported and measured weight (1.26 kg) and BMI (0.478 kg/m^2^) were relatively small. Females under-reported their weight and the calculated BMI from estimated weight and height was underestimated by adolescents with overweight/obesity. The impact of media use on the adolescent’s perception of being thin relative to the accuracy of the calculated BMI was significant (p = 0.043–0.001). The age-adjusted odds ratio of the calculated (underestimated) BMI in adolescents without overweight/obesity was 0.437 (95% CI = 0.257–0.741; p = 0.002). It was concluded that the validity of self-reported weight was high. Adolescents with overweight/obesity were more likely to underestimate their weight and calculated BMI. Educating adolescents about proper lifestyles and weight loss through media appears warranted.

## Introduction

Anthropometric measurements, including weight, height, and body mass index (BMI), are often used for assessing the physical growth, nutritional status, and overall health of children and adolescents^1^. Although BMI is considered a crude estimate of excess body weight, it is an internationally accepted obesity assessment method, as it generally correlates well with body fat in the population^2,3^. It is well-recognized, though, that excess body weight is largely associated with non-communicable diseases such as cardiovascular illness, type 2 diabetes mellitus, and hypertension^3^. Objective measurements of weight and height are usually desired when calculating BMI; however, due to time, financial resources, and manpower constraints^4^, self-reported weight and height are commonly used in large epidemiology surveys and surveillance systems to generate regional and national estimates of overweight and obesity^5,6^. Decision makers often use such information to allocate resources and set priorities in health care systems^7^.

Although self-reported body weight and height measures are subject to inaccuracy, which can result in incorrect BMI classification, self-reported measurements of body weight and height have been used to obtain estimative of important parameters in some epidemiological surveys. In a large national surveillance in Brazil, the mean agreements between self-reported and measured weight and height were 99.6% and 100.6%, respectively^8^. Also, results from the School Physical Activity and Nutrition surveillance system in Texas, USA, which included more than 24,000 students in the 8th and 11th grades, indicated that when direct measurement of weight and height is not practical, self-reported assessment offers a reliable proxy measure in adolescents^9^. Some studies, however, have identified a greater discrepancy in self-reported and measured height and weight among certain groups, including overweight or obese individuals compared to healthy or underweight individuals^10–14^. An increasing number of children and adolescents worldwide are overweight, and the first step in preventing obesity is to identify those individuals at risk of becoming overweight. Mexican children and adolescents 10–16 years old, who were diagnosed with overweight/obesity, revealed that the barriers to losing weight from the perspective of the children and their parents included lack of perception of being overweight and its identification as a disease and its consequences^15^. It is therefore surprising that not many studies have investigated the discrepancy between self-reported and measured body weight and height in the Arab countries^10,16^. Indeed, to the authors’ best knowledge, the accuracy of self-reported weight and height among Kuwaiti adolescents has not been previously reported.

Factors associated with misperception of body weight are many and include age, sex, ethnicity, socio-economic status, BMI status, body-size perception and weight status, pubertal status, place of residence, physical activity, smoking, sedentary behaviors, and vegetable and fruit consumption^17–19^. However, the pressure from mass media (television, magazines, and social media) on body weight concerns is usually enormous, especially among young people. Young women who spent time on Facebook, were reported to be in a more negative mood than those who were exposed to the control website^20^. In addition, a study showed that such media pressure on female university students from five Arab countries led to a greater risk of dieting to lose weight and changed their ideas of a perfect body shape^21^. A cross-sectional survey that was conducted on Jordanian school adolescents aged 15–18 years found that most adolescents with or without obesity had shown a high impact of reading magazines on their dieting to lose weight^22^. In a sample of 17- to 32-year-old university students from five Arab countries, exposure to television had a weaker association than exposure to magazines regarding females’ body weight concerns^21^. In addition, the majority of the American preadolescent and adolescent girls enrolled in 5th through 12th grades were unhappy with their body weight and shape, and this was strongly associated with the frequency of reading fashion magazines^23^. Further, an Australian study involving adolescents enrolled in Grades 7 and 9 showed that the major influences on weight loss for girls were pubertal status and exposure to the media^24^.

Therefore, the purpose of the present study was to investigate the accuracy of self-reported body weight and height among Kuwaiti adolescents in comparison with the measured values and to examine the associations of self-reported weight and the calculated BMI from the estimated weight and height with the impact of media use on adolescents’ decision to lose weight and their perception of body thinness.

## Results

A total of 706 adolescents (363 females) between the ages of 15 and 18 years were included in the study. A very high response rate was obtained (above 95%). Based on measured weight and height, nearly 50% of the participants were overweight or obese. Approximately 33% of the participants did not report their weight and about 40% did not report their height. There were no significant differences (*p* values ranged from 0.071 to 0.994) in the responses to media-related variables between adolescents who reported their weight and height data and those who were missing their weight and height information. Results of ICC as a measure of absolute reliability indicated that the agreement between estimated (self-reported) and measured weight and height were high. (95% CI) were 0.973 (0.968–0.977) and 0.867 (0.839–0.891), respectively. The ICC between measured and the calculated BMI from estimated weight and height (95% CI) was 0.713 (0.647–0.766). Pearson correlation coefficients between measured and estimated weight, height, and BMI were 0.947, 0.777, and 0.623, respectively.

**Figure 1:**
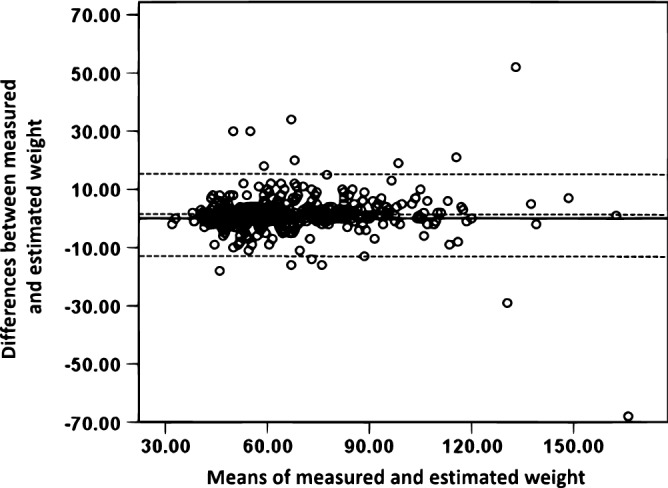
Bland and Altman levels of agreement plot for the measured and estimated body weight. The centered dotted line represents the mean differences or bias (1.26 kg) and the outer doted lines represent the upper (14.2 kg) and lower (− 11.7 kg) 95% of limits of agreement.

**Figure 2:**
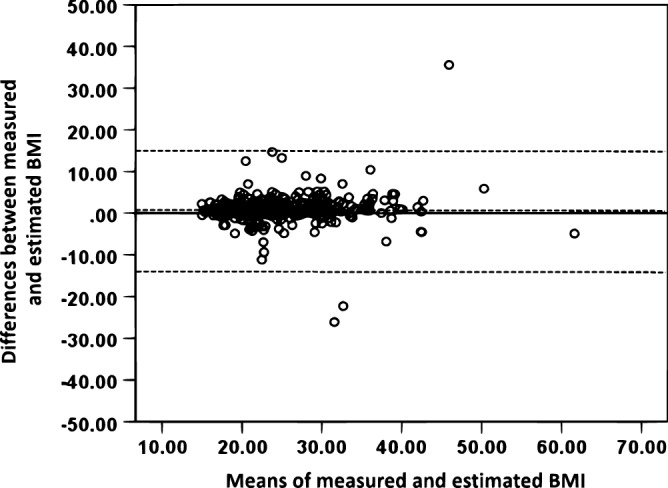
Bland and Altman levels of agreement plot for the actual and calculated BMI from self-reported weight and height. The centered dotted line represents the mean differences or bias (0.478 kg/m^2^) and the outer doted lines represent the upper (17.5 kg/m^2^) and lower (− 17.0 kg/m^2^) 95% of limits of agreement.

Table 1 shows the descriptive characteristics of the participants relative to gender. There was no significant difference (p = 0.466) in chronological age between male and female adolescents. However, there were significant gender differences between males and females in the reported age at pubertal maturation (p < 0.001), reported and measured body weight (p < 0.001) and height (p < 0.001), and estimated (p = 0.002) and measured (p = 0.031) BMI values. Moreover, Bland–Altman plots (Figs. 1 and 2) indicated that the mean differences between self-reported and measured weight and corresponding BMI were relatively small for the total sample (1.26 kg and 0.478 kg/m^2^, respectively). The majority of values fell within the upper and lower limits of agreement (± 2 SD), with random scatter plots and small systemic bias detected.

**Table 1:** Descriptive characteristics of the participants relative to gender.

Variable	All N = 706	Males N = 343	Females N = 363	*p*-value*
Age	16.5 ± 0.94	16.5 ± 0.89	16.5 ± 0.98	0.466
Age at maturation	12.9 ± 1.3	13.3 ± 1.2	12.6 ± 1.3	**< 0.001**
Estimated body weight by the participant (kg)	65.5 ± 20.4	75.5 ± 23.1	57.4 ± 13.2	**< 0.001**
Measured body weight (kg)	69.3 ± 22.4	77.1 ± 24.0	61.9 ± 17.4	**< 0.001**
Estimated height by the participant (cm)	163.6 ± 11.1	171.3 ± 11.4	158.1 ± 6.7	**< 0.001**
Measured height (cm)	162.7 ± 9.4	169.8 ± 7.1	155.9 ± 5.6	**< 0.001**
BMI from estimated weight and height (kg/m^2^)	25.1 ± 11.1	27.3 ± 15.5	23.3 ± 5.0	**0.002**
BMI from measured weight and height (kg/m^2^)	25.9 ± 7.4	26.6 ± 7.9	25.4 ± 6.7	**0.031**

Data are means ± standard deviations or percentage.

*T test for independent samples or Chi squares tests for the proportion. Significant values are in bold.

Table 2 presents the differences between measured and reported body weight, height, and BMI, relative to gender. No significant differences were observed between the measured and estimated weight, height, or BMI. Girls were significantly (p = 0.038) more likely to underreport their weight and hence obtain lower calculated BMI from the estimated weight and height values (not shown in table). There were no significant differences (p = 0.589) in the proportions of correctly calculated BMI from the estimated body weight and height in relation to gender (males = 4.9% and females = 5.8%). The findings also showed that self-reported age at maturation was not associated with self-reported weight, height, or with the degree of underestimation or overestimation of body weight or calculated BMI.

**Table 2:** Differences between measured and reported body weight, height and corresponding calculated BMI.

Variable	All	Males	Females	*p*-value*
Differences between measured and estimated weight (kg)	1.26 ± 6.6	0.68 ± 7.2	1.73 ± 6.0	0.086
Differences between measured and estimated height (cm)	− 1.03 ± 6.9	− 0.47 ± 9.2	− 1.43 ± 4.8	0.215
Differences between measured and estimated BMI (kg/m^2^)	0.48 ± 8.7	− 0.56 ± 12.4	1.30 ± 3.5	0.065
**Accuracy of BMI based on body weight and height estimation** (%)				
Overestimation	24.7	26.5	23.3	0.745
Correct estimation	5.4	4.9	5.8	
Underestimation	69.9	68.6	70.9	

* T-test for independent samples or Chi Squares tests for the proportion.

The accuracy of calculating BMI based on body weight and height estimation in relation to overweight or obesity status is displayed in Table 3. There was a significant difference (p = 0.028) in the accuracy of calculating BMI based on weight and height estimation by the Kuwaiti adolescents relative to overweight/obesity or non-overweight/non-obesity status. Adolescents with overweight or obese seemed to greatly underestimate their body weight and thus had lower calculated BMI from estimated weight and height values.

**Table 3:** Accuracy of estimating body weight relative to overweight or obesity status.

Variable	Non-overweight or non-obesity (BMI < 25 kg/m^2^)*	Overweight or obesity (BMI ≥ 25 kg/m^2^)*	*p*-value*
Overestimation	30.4	18.4	**0.028**
Correct estimation	5.2	5.7	
Underestimation	64.4	75.9	

*Overweight or obesity classification was based on the extended International Obesity Task Force (IOTF) age- and sex-specific BMI cutoff reference standards, which is equivalent to adults’ BMI at above or below 25 kg/m^2^ (Ref.^26^). Significant values are in bold.

The influences of selected variables related to media on the accuracy of calculated BMI from body weight estimation by the Kuwaiti adolescents are shown in Table 4. The impact of reading newspapers (p < 0.001) or surfing the Internet (p = 0.002) on the adolescents’ decision to lose weight was significant. Also, the impact of the media on the adolescents’ perception to be thin relative to the accuracy of BMI calculation from the estimated weight and height was significant (p values ranged from 0.043 to 0.001). Those adolescents who were strongly influenced by the media to be thin appeared to underestimate their calculated BMI from their estimated weight and height. Finally, the results of the logistic regression analysis, adjusted for age, for selected variables relative to underestimation or overestimation of calculated BMI among Kuwaiti adolescents are shown in Table 5. The only significant difference between underestimation and overestimation of calculated BMI from estimated weight and height was overweight or obesity status. The age-adjusted odds ratio for adolescents without overweight or obesity when calculating BMI from their body weight estimation was 0.437 (95% CI = 0.257–0.741; p = 0.002).

**Table 4:** Impacts of selected media-related variables on the accuracy of BMI calculation based on self-report body weight and height by the Kuwaiti adolescents.

Variable	Accuracy of BMI calculation (%)			*p*-value*
	Overestimated	Correct estimation	Underestimated	
**Reading newspapers influenced my decision to lose weight**				**< 0.001**
None	4.4	25.0	3.1	
Weak	1.1	0.00	0.4	
Moderate	0.0	0.0	0.0	
Strong	94.5	75.0	96.5	
**Viewing TV programs influenced my decision to lose weight**				0.096
None	5.5	10.0	3.1	
Weak	1.1	0.0	0.0	
Moderate	38.5	40.0	52.9	
Strong	54.9	50.0	44.0	
**Surfing the internet influenced my decision to lose weight**				**0.002**
None	4.4	20.0	2.3	
Weak	1.1	0.0	0.0	
Moderate	34.1	40.0	42.0	
Strong	60.4	40.0	55.7	
**Reading newspapers influenced my perception about thinness**				**0.006**
Not at all	8.8	30.0	6.3	
Little	63.7	55.0	66.8	
A lot	27.5	15.0	27.0	
**Viewing TV programs influenced my perception about thinness**				**0.001**
Not at all	5.5	25.0	3.1	
Little	31.9	20.0	31.9	
A lot	62.6	55.0	65.0	
**Surfing the internet influenced my perception about thinness**				**0.043**
Not at all	6.6	15.0	2.3	
Little	28.6	30.0	32.7	
A lot	64.8	55.0	65.0	

* Chi Squares tests for the proportion.

Significant values are in bold.

**Table 5:** Logistic regression analysis, adjusted for age, for selected variables related to underestimation or overestimation of the calculated BMI among Kuwaiti adolescents.

Variable	Overestimation versus underestimation of calculated BMI*			
	aOR	(95% CI)	SEE	*p-*value
Age	1.081	0.829–1.410	0.135	0.564
**Gender (girl = ref)**	1.00			
Boys	0.747	0.448–1.248	0.261	0.266
**Overweight or obesity (overweight/obesity = ref)**	1.00			
Non-overweight or non-obesity	0.437	0.257–0.741	0.270	**0.002**
**Influence of TV use on the decision to lose weight (strong = ref)**	1.00			
Moderate	1.760	0.958–3.234	0.310	0.068
None/seldom or weak**	0.667	0.139–3.207	0.801	0.613
**Influence of Internet use on the decision to lose weight (strong = ref)**	1.00			
Moderate	1.027	0.542–1.947	0.326	0.935
None/seldom or weak**	0.887	0.163–4.831	0.865	0.890
**Influence of TV use on the perception about thinness (A lot = ref)**	1.00			
Little	0.804	0.453–1.428	0.293	0.457
Not at all	0.717	0.172–2.986	0.728	0.648
**Influence of Internet use on the perception about thinness (A lot = ref)**	1.00			
Little	1.185	0.642–2.186	0.312	0.587
Not at all	0.351	0.091–1.361	0.691	0.130

*Overestimation was used as a reference category. *aOR* adjusted odds ratio, *CI* confidence interval, *ref* reference category, *SEE* standard error.

**Categories were combined due to redundancy. Significant values are in bold.

## Discussion

The present study investigated the accuracy of self-reported body weight and height among Kuwaiti adolescents and its associations with overweight or obesity. It also examined the associations of self-reported weight and BMI with the impact of media use on adolescents’ decision to lose weight and their perception of body thinness. The major findings showed that the reliability coefficient of self-reported weight was excellent (ICC = 0.973). Further, Bland–Altman plots indicated that the mean difference between self-reported and measured weight and the corresponding BMI were fairly small for the total sample (1.26 kg and 0.478 kg/m^2^, respectively), with random scatter plots and small systemic bias detected. Females were significantly more likely to under report their weight than males and hence obtain lower calculated BMI from estimated weight and height values. However, there were no significant differences in the proportions of correctly calculated BMI relative to gender. Moreover, adolescents with overweight or obesity, and those who were strongly influenced by the media to be thin, appeared to underestimate their calculated BMI from their estimated body weight and height. Overweight or obesity status was associated with under-estimation of adolescents’ body weight and calculated BMI.

Self-reported weight and height are commonly used in large epidemiological surveys and surveillance to create regional and national estimates of overweight and obesity, especially when direct measurements are not possible due to restrictions in budget and human resources^5,6^. Numerous studies have shown that self-reported weight and height may be acceptable^5,9,18,25^. However, the validity of self-reported weight and height among adolescents needs to be verified, as misreporting could lead to misclassification of BMI and therefore overestimation or underestimation of the burden of BMI-related diseases^12,26–28^. Self-reported weight and height data are not without some shortcomings. Missing self-reported data was raised as a problem in the Scottish Health Behaviour in School-Aged Children survey; nearly 59% of self-reported weight data was missing, with girls self-reporting less than boys^29^. Adolescents who reported low physical activity, low vegetable consumption, and high computer gaming on weekdays were more likely to not report their weight^29^. A study from Jeddah, Saudi Arabia, reported that more than half of the school children participants were unaware of their body weight and did not report such weight^10^. In the present study, approximately 30% of the Kuwaiti adolescents did not report their weight, and there were no significant differences in media-related variables between those adolescents reporting and not reporting their weight and height information.

In the current study, the value of absolute reliability between self-reported and measured body weight (0.973), as measured by the ICC coefficient, was considered excellent^30^. A similarly high ICC coefficient value between self-reported and direct measurement of body weight was reported for healthy school adolescents (0.96), and the mean difference between self-reported and direct measurement of weight, based on Bland–Altman plots, was relatively small^18^. Results of the Bland–Altman plot in the current study showed a small bias and a good agreement between self-reported and direct anthropometric measurements. The present study showed that the ICC between calculated BMI from measured and estimated height and weight was 0.713. In addition, based on Bland–Altman levels of agreement analysis, the mean difference between self-reported and measured weight and corresponding BMI were fairly small for the total sample (1.26 kg and 0.478 kg/m^2^, respectively), with random scatter plots and small systemic bias detected. Among American students from 8th to 11th grade, the differences in self-reported versus objectively measured height and weight resulted in underestimation of BMI ranging from − 0.23 kg/m^2^ among White boys to − 0.7 kg/m^2^ among African-American girls^9^. In a recent study conducted on young adults between 18 and 30 years, classification of BMI from self-reported and actual measurements showed that 88% of participants were placed in the same BMI category^25^. BMI values based on self-reported weight and height are described to be inaccurate for BMI prediction at an individual level^12^.

The results of the present study regarding the mean differences between self-reported and measured body weight and height were close to those reported in a study with Malaysian school adolescents^18^. However, the mean differences found in our study are much lower than those reported in studies with New Caledonian school-going adolescents^17^ and Saudi school children^10^. The results of this study are also similar to those found in a large surveillance with American students from 8 to 11th grade, in which adolescents overestimated their height and underestimated their weight^9^. Furthermore, our correlation coefficients between measured and self-reported anthropometric variables were similar, in direction and magnitude, to those reported by a recent study conducted on young Australian adults 18–30 years of age^25^.

Our findings showed significant gender differences between males and females in the self-reported and measured body weight, height, and calculated BMI from estimated weight and height values. Kuwaiti girls were significantly more likely to underreport their weight and hence end up with lower calculated BMI values. Elsewhere, the accuracy of anthropometric reporting by American female high school students was significantly higher than that of males^31^. Compared to boys in all age groups, differences between self-reported and directly measured weight, height and BMI were greater among Estonian adolescent girls^32^. than Among Australian youth, more females (70%) than males (35%) underreported their weight^33^. Also, female Saudi school children were observed to underestimate their body weight more than males^10^. However, the self-reported bias in body weight among Greek high school children did not differ relative to gender^19^.

Our study showed that nearly 70% of the Kuwaiti adolescents underreported their body weight to some degree. This finding of underreporting of body weight among adolescents is in agreement with previous studies conducted around the world^9,18,19,27,33^. However, the current study found a significant difference in the accuracy of calculating BMI based on weight and height estimation by the Kuwaiti adolescents relative to overweight or obesity status. Adolescents with overweight or obesity tended to underestimate their body weight and thus had lower calculated BMI values. Such findings have been substantiated in the previous literature. Underweight American high school students, underreported height and overreported weight, while overweight and obese students overreported height and underreported weight^31^. However, the differences between measured and estimated body weight among Greek high school students were higher among heavier students^19^. Also, obese Korean high school students, tended to underestimate their weight and BMI and overestimate their height more than non-obese adolescents^26^. In addition, the German Health Interview and Examination Survey for Children and Adolescents indicated that self-reported and measured height and weight can lead to inaccurate estimates of the prevalence of under- and overweight status^27^. Further, Jordanian adolescents with overweight or obesity were more likely to underestimate their weight and that having a BMI within the overweight/obesity category was a strong predictor of inconsistent weight perception^16^. Other studies also showing underreporting their weight include Australian youth^33^ and Malaysian adolescents^18^.

It is well recognized, however, that the prevalence of overweight or obesity based on self-reported data should be interpreted with caution because it could underestimate the true prevalence. It is not recommended to use self-reported data, especially in clinical settings, when assessing and monitoring an individual’s nutritional status for diagnosis of malnutrition and recommendation of nutritional intervention^4^. The magnitude of discrepancy between self-reported and measured weight and height and the corresponding BMI could be related to many potential factors, including age, sex, ethnicity, socio-economic status, sexual maturation, parental overweight, place of residence, and many lifestyle behaviors^17–19,27^. Also, parental health behaviors were reported to play a significant role in adolescents' body weight^34^. In the present study, self-reported age at maturation was not associated with self-reported weight, height, or degree of underestimation or overestimation of body weight or calculated BMI.

In recent years, social media marketing has become a preferred marketing strategy for a range of industries, including the food industry^35^, and the increase in social media marketing and communications that target children and adolescents has raised some major concerns^20,36^. Our findings showed that the impact of reading newspapers or surfing the Internet on adolescents’ decision to lose weight was significant, especially among those who underestimate or overestimate their calculated BMI from estimated body weight and height. Also, the media impact on the adolescents’ perception of being thin relative to the accuracy of the BMI calculation from estimated weight and height values was significant, particularly among those adolescents who underestimated or overestimated their calculated BMI. Different kinds of media may have different impacts on adolescents’ body weight-related aspects. For example, young university students from five Arab countries who were exposed to television had a weaker association than exposure to magazines regarding females’ body weight concerns^21^. Key indicators of eating disorders were significantly more prevalent among Fijian schoolgirls following prolonged exposure to television^37^. Among Jordanian school adolescents, most students with or without obesity had shown a high impact of reading magazines on their dieting to lose weight^22^. Furthermore, the majority of the American preadolescent and adolescent girls, who enrolled in 5th to 12th grade, were unhappy with their body weight and shape, and this was strongly associated with the frequency of reading fashion magazines^23^. Saudi adolescent girls exposed to television advertisements were more likely to consume dessert and have more attempts at losing weight^38^. Also, among American adolescents, media influence to lose weight was related to more frequent unhealthy weight control behaviors among boys with a BMI z-score of 1.23 or higher and among girls with low to average levels of global self-worth^39^.

The present study had several limitations. First, body weight is affected by many factors, including physical activity, sleep, and dietary habits. We did not assess any of these variables. Second, body weight, and hence BMI, is also affected by muscular status, especially among males who start engaging in body building activities at this age. This is another factor that we did not assess. Third, the survey used in the current study was subject to recall bias and social desirability effects. Fourth, the cross-sectional nature of the study does not imply causality of media impacts on overestimation or underestimation of body weight. Fifth, nearly 30% of our participants did not self-report their body weight. However, there were no significant differences in the responses to media-related variables between adolescents reporting their weight and height data and those who were missing their weight and height information. Sixth, in our questionnaires, unfortunately, we did not differentiate between mass media and social media. Despite these limitations, our results are consistent with several international studies suggesting that self-reporting of weight and height can be an acceptable method for assessing adolescent body weight and height. The results of the present study may be helpful in planning and developing novel surveys focused on health and anthropometric data.

## Conclusion

The present study investigated the accuracy of self-reported body weight and height among Kuwaiti adolescents and its associations with overweight or obesity and examined the associations of self-reported weight and the calculated BMI from estimated weight and height with the impact of media use on adolescents’ decision to lose weight and their perception of body thinness. The major findings showed that the reliability coefficient of self-reported weight was excellent (ICC = 0.973). Further, Bland–Altman plots indicated that the mean difference between self-reported and measured weight and the corresponding BMI from estimated weight and height were fairly small for the total sample (1.26 kg and 0.478 kg/m^2^, respectively), with random scatter plots and small systemic bias detected. Females were significantly more likely to underreport their weight than males and hence end up with lower calculated BMI from estimated weight and height values. However, there were no significant differences in the proportions of correctly calculated BMI from estimated weight and height relative to gender. Moreover, adolescents with overweight or obesity appeared to underestimate their calculated BMI. Those adolescents who were strongly influenced by the media to be thin appeared to underestimate their calculated BMI from estimated weight and height. Overweight or obesity status was associated with underestimation of adolescents’ body weight and the corresponding calculated BMI. Compared to adolescents with overweight or obesity, the age-adjusted odds ratio of underestimated (calculated) BMI from weight and height in adolescents without overweight or obesity was 0.437 (95% CI = 0.257–0.741; p = 0.002). Educating adolescents about proper nutrition, physical activity, and weight loss through media appears warranted.

## Methods

### Sample selection

This cross-sectional study randomly selected Kuwaiti secondary students between the ages of 15 and 18 years using a multistage stratified sampling technique. Girls’ and boys’ public and private schools were used for selecting the sample based on geographical/administrative locations (governorates) in the State of Kuwait. One public boys’ school and one public girls’ school were selected from each governorate with a total of 10 schools. The data collection was conducted during the school year 2019. Additional two private boys’ schools and two private girls’ schools were also selected, totaling 14 public and private schools. In each school, one classroom from each grade (i.e., 10th, 11th, and 12th) was randomly chosen. A total sample of 343 boys and 363 girls was recruited for the study. Ethical approval was obtained from the Research Committee, College of Health Sciences, Public Authority for Applied Education and Training, Kuwait. The research procedures were conducted in accordance with the principles expressed in the Declaration of Helsinki. Informed consents were obtained from all participating students and consents were attained from their parents if they were younger than 18 year-olds. The Kuwait Ministry of Education approved the study.

### Measurements

Body weight was measured to the nearest 100 g (Seca 875 Weight Scale) and height to the nearest 0.1 cm (Seca 213 Standiometer) by trained researchers. All measurements were conducted inside the schools with minimal clothing and without shoes. BMI was computed as the ratio of weight in kilograms divided by the squared height in meters. The extended International Obesity Task Force (IOTF) age- and sex-specific BMI cutoff reference standards were used to classify underweight, normal weight, and overweight or obesity relative to the adolescent’s age^40^. The participants were also asked to self-report their body weight and height. Further, male and female adolescents were asked to report their age at semenarche and menarche and used as indicator of age at maturation.

A previously used, specifically designed self-report questionnaire^21^ was utilized to obtain information on media use (television and Internet use and several questions on the impact of media on dieting to lose weight and the perception of body thinness). Examples of the questions include: How often do you read the newspapers, watch television, or use the Internet per day? Does reading the newspapers, watching television, or surfing the Internet influence you to lose weight? Does reading the newspapers, watching television or using the Internet influence your perception about body thinness?

### Statistical analysis

Data were entered into an SPSS data file, checked, cleaned, and analyzed using IBM-SPSS program, version 22 (Chicago, IL, USA). Descriptive statistics were obtained for all variables and reported as means and standard deviations or percentages. Intraclass correlation coefficient (ICC) was used to test the absolute reliability of self-reported weight and height with a two-way mixed effects model. According to Shrout and Fleiss's classification, ICC values greater than 0.75 indicate excellent reliability, values between 0.04 and 0.75 indicate fair to good, and values less than 0.40 indicate poor reliability^30^. However, other references for the ICC coefficient consider values between 0.75 and 0.9 as indicating good reliability and values greater than 0.90 as indicating excellent reliability^41^.

The Bland–Altman limits of agreement were computed, and the mean differences (bias) as well as the 95% upper and lower limits of agreement plotted for differences between measured and estimated body weight or calculated BMI against means of measured and estimated weight or BMI^42^. Normality of the distribution of differences was tested using histograms. Differences between males and females in anthropometric measurements were tested using a *t* test for independent samples. Chi-square tests of proportions were used to test differences in selected variables related to the influence of media on adolescents’ decision to lose weight or their perception of body thinness according to overweight or obesity status. Two-way ANOVA (gender by age category) was used to test differences in the calculated BMI across ages and genders, with Bonferroni test used for correcting multiple testing. Logistic regression analysis, adjusted for age, was used to test differences in selected variables in relation to underestimation or overestimation of the calculated BMI from self-reported weight and height among Kuwaiti adolescents. If measured BMI was higher than the calculated BMI from estimated weight and height values, it was considered an underestimated BMI value, and if the measured BMI was lower than the estimated BMI, it was considered overestimated BMI value. The adjusted odds ratio (aOR) and confidence intervals were reported. The alpha level was set at 0.05, and a *p*-value less than the alpha level was considered significant.

## Data Availability

All data generated or analyzed during this study are included in this published article. Any additional data will be available from the corresponding author upon reasonable request.
